# Mosaic 22q11.2 microdeletion syndrome: diagnosis and clinical manifestations of two cases

**DOI:** 10.1186/1755-8166-1-18

**Published:** 2008-08-10

**Authors:** Ashutosh Halder, Manish Jain, Madhulika Kabra, Neerja Gupta

**Affiliations:** 1 Department of Reproductive Biology, All India Institute of Medical Sciences, New Delhi, India; 2 Department of Pediatrics, Pediatrics Genetics Unit, All India Institute of Medical Sciences, New Delhi, India

## Abstract

Chromosome 22q11.2 microdeletion syndrome is due to microdeletion of 22q11.2 region of chromosome 22. It is a common microdeletion syndrome however mosaic cases are very rare and reported only few previous occasions. In this report we describe two unrelated male children with clinical features consistent with 22q11.2 microdeletion syndrome characterized by cardiac defect, facial dysmorphism and developmental deficiency. One of the cases also had trigonocephaly. Interphase & metaphase FISH with 22q11.2 probe demonstrated mosaicism for hemizygous deletion of 22q11.2 region. Mosaicism is also observed in buccal cells as well as urine cells. Parents were without any deletion. These two cases represent rare cases of mosaic 22q11.2 microdeletion syndrome.

## Background

The 22q11.2 microdeletion syndrome is the most common microdeletion syndrome with an estimated incidence of one in 4000 births [1]. It has a wide phenotypic spectrum. Almost all cases result from a common deletion of chromosome 22q11.2 locus. Diagnosis of this microdeletion syndrome is based on prometaphase banding cytogenetics [2], fluorescent in situ hybridization (FISH) [3], array comparative genomic hybridization (aCGH) [4], quantitative fluorescent polymerase chain reaction (QFPCR) [5] with polymorphic micro satellite marker and multiplex ligation dependent probe amplification (MLPA) [6]. Interphase FISH is the prime method for diagnosis of mosaicism. Interphase FISH, being the only way to analyze large number of cells individually & quickly, has the ability to diagnose mosaicism including low level very easily and reliably. Diagnosis of 22q11.2 microdeletion mosaicism through FISH on amniocytes & cord blood cells [7], on peripheral blood lymphocytes [8,9] and on cardiac tissue [9] have been reported. In this report we describe two male children with clinical features of 22q11.2 microdeletion syndrome and FISH analysis showed mosaicism for a deletion in the critical region (22q11.2) in peripheral blood nucleated cells (both metaphase & interphase cells). Furthermore, one of the children had trigonocephaly (premature closure of metopic suture) in addition to features of 22q11.2 microdeletion syndrome despite low level of deleted cells. Trigonocephaly was reported only once in the literature [10]. We here report two rare cases of mosaic 22q11.2 microdeletion syndrome.

## Case report

### Case 1

A two years and ten months male child was referred from Fateyabad, Haryana, India to our hospital for cardiac malformation (tetralogy of Fallot) and facial dysmorphism. Pediatric geneticist referred the child to us to evaluate for 22q11.2 microdeletion syndrome. The child was born to a 23 year-old mother at 33 weeks 5 days by vaginal delivery in a local private hospital. He was 1400 gm at birth and was in nursery for first 20 days after birth. He was first born child of the couple. His record of length and head circumference was not available. There was no history of antenatal complications. Suckling was defective throughout infancy for which he was spoon fed until one year of age and never breast fed. He had recurrent episodes of upper respiratory infection including fever since infancy for which he was admitted several times in private hospital for the treatment. His milestone was delayed; unable to speak, walk or stand up even at age of 2 years ten months. He has no dyspnoea or cyanosis. There was no similar problem in the family excepting squint in mother and beta thalassaemia major in paternal side.

**Figure 1:**
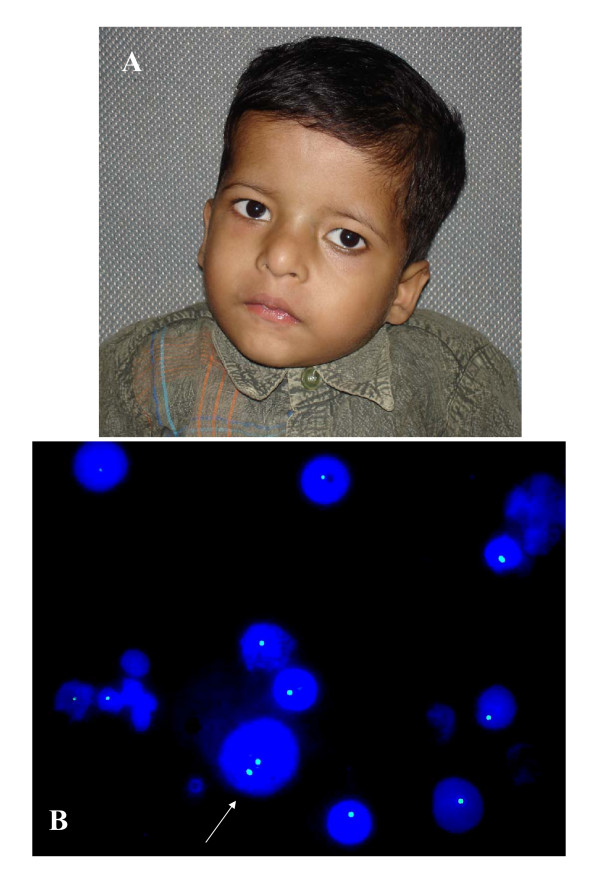
**A is showing broad nose, square shaped tip of nose, small philtrum, hypertelorism, telecanthus, squint and low set ears.** B is showing 22q11.2 FISH with deletion (most cells) and without deletion (arrow) on interphase cells obtained from peripheral blood.

Physical examination revealed dysmorphic features & generalized hypotonia. He had short & broad nose, small mouth, down turn upper lip, hypertelorism, telecanthus and squint (Fig. 1A). Ears were low set, deficient in vertical diameter and dysplastic. Palate was high arched. Hands & fingers were long and slender. His weight was 9 kg and height was 75.5 cm (both below 3^rd ^percentile). Head circumference was 45 cm (below -2SD/below 2^nd ^percentile), however proportionate to height & weight. His developmental quotient was between 41–45% of expected. Ophthalmologic examination was revealed squint. Extensive cardiovascular work up including echocardiography revealed mild pulmonary stenosis, large malaligned ventricular septal defect, dilated aortic root and was suggestive of tetralogy of Fallot. CT scan of head & brain was normal. There was no hypocalcemia. Conventional cytogenetics from lymphocyte culture was normal.

Since the patient had findings strongly suggestive of 22q11.2 microdeletion syndrome FISH study to detect a possible deletion in the critical 22q11.2 region was done using PAC/BAC clones specific for 22q11.2 locus (RP5-882J5 & CTA-154H4 obtained from Uniba Biologia, Italy, by curtsy of Prof. M Rocchi). Interphase FISH was done using 1 ml of blood obtained from the patient. Blood nucleated cells washed in phosphate buffer saline solution three times before 30 minutes hypotonic treatment (50 mMol KCL) and fixation in methanol:acetic acid solution (3:1 ratio). Cells re-suspended in 100 ul fresh fixative. Approximately 20 ul cell suspension was used to prepare a slide. PAC/BAC clones were grown in LB broth, DNA extracted and about 1 ug DNA was labeled with green flurochrome (FITC) or red flurochrome (Cy3) by nick translation method. About 100 ng labeled probe was used for FISH. FISH analysis was carried out using Olympus BX51 microscope with epifluorescence attachment and image was captured through spectral imaging system. A total of 1312 interphase nuclei were scored. Interphase FISH result showed 1100 (83.8%) nuclei with hemizygous deletion for 22q11.2 locus and 210 (16%) nuclei with normal diploid state (Fig. 1B). Normal control cases displayed two signals in approximately 98% nuclei whereas positive controls displayed hemizygous deletion in approximately 98% nuclei. This finding was conclusive for mosaic 22q11.2 microdeletion with presence of low level of normal cells. FISH on metaphase spread pick up deletion in all excepting one of 25 metaphases studied. Interphase FISH was also carried out on buccal cells and urinary cells as described before [11] to find out whether mosaicism restricted to blood or generalized. Mosaicism was confirmed in all three types of cells (Table 1). Parents were also screened for deletion and results were negative.

**Table 1:** Details of FISH results of cases and their parents

**Parameters**	***One Signal*** *** (%)***	**Two Signals** ** (%)**	**Other Signals** ** (%)**	**Total** ** Cells**	**Remarks**
**Patient 1**					
Blood Interphase	1100 (83.8)	0210 (16)	0002 (0.2)	1312	16% normal cells
Blood Metaphase	0024	0001	0000	0025	One normal cell (4%)
Buccal Cells	0014 (77.8)	0004 (22.2)	0000	0018	22.2% normal cells
Urinary Cells	0008 (88.9)	0001 (11.1)	0000	0009	11.1% normal cells
Mother's Blood (interphase)	0000	0100	0000	100	Normal
Father's Blood (interphase)	0001	0081	0001	0083	Normal
					
**Patient 2**					
Blood Interphase	121 (11.6)	925 (88.4)	000	1046	88.4% normal cells
Blood Metaphase	006 (15)	034 (85)	000	040	85% normal cells
Buccal Cells	008 (13.8)	050 (86.2)	000	058	86.2% normal cells
Urinary Cells	007 (14.6)	041 (85.4)	000	048	85.4% normal cells
Mother's Blood (metaphase)	000	030	000	030	Normal
Father's Blood (metaphase)	000	020	000	020	Normal

### Case 2

A one and half year old male child was referred from a village near Sirsa, Haryana, India to our hospital for cardiac malformation and facial dysmorphism. Pediatric geneticist referred the child to us to evaluate for 22q11.2 microdeletion syndrome. The child was born to a 27 year-old mother at term by vaginal delivery at local hospital. He was 1750 gm at birth. He was second born child of the non-consanguineous couple and his elder 3 years old sister was normal. His record of length and head circumference at birth was not available. There was no history of antenatal complications. However, early neonatal period was complicated by unconjugated hyperbillirubinemia that was managed by phototherapy. Suckling was normal however prone to have recurrent episode of vomiting in later part of infancy following intake of foods other than milk. His milestone was grossly delayed. He has significant dyspnoea since last six to seven months. There was no similar problem in the family in either side.

**Figure 2:**
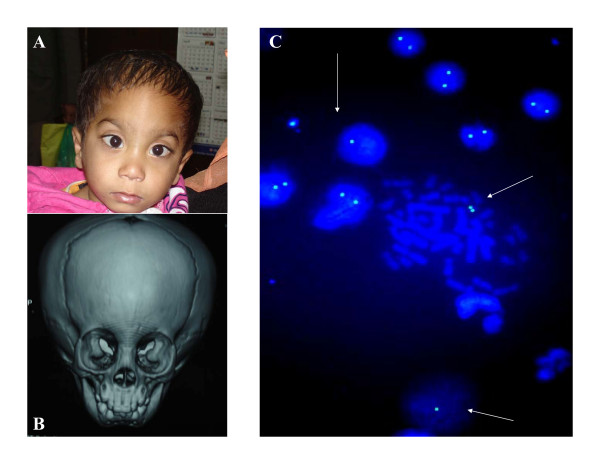
**A is showing broad & short nose, small moth, wide philtrum, thin upper lip, hypertelorism, telecanthus, upward slanting almond shaped eyes, low set ears and forehead prominence.** B is CT scan of skull showing fusion of metopic suture. C is showing 22q11.2 FISH on lymphocytes (metaphase & interphase cells) without and with deletions (arrow).

Physical examination revealed dysmorphic features & generalized hypotonia. Most prominent feature was trigonocephaly with metopic prominence leading to forehead prominence. His head circumference was 45 cm (below -2 SD/below 2^nd ^percentile). He had short & broad nose, small mouth, wide philtrum, thin upper lip, hypertelorism, telecanthus and upward slanting almond shaped eyes (Fig. 2A). Ears were low set. Ophthalmologic & auditory examination revealed no abnormality. He had central cyanosis and significant clubbing of fingers. Extensive cardiovascular work up including echocardiography was suggestive of tetralogy of Fallot with reversal of flow. An X ray skull and CT scan (Fig. 2B) of head & brain was consistent with craniosynostosis (due to premature metopic suture fusion). There was no hypocalcemia. Conventional cytogenetics from lymphocyte culture was normal.

Since the patient had findings suggestive of 22q11.2 microdeletion syndrome FISH study was done using PAC/BAC clones specific for 22q11.2 locus (RP5-882J5 & CTA-154H4, obtained from Uniba Biologia, Italy, by curtsy of Prof. M Rocchi). A total of 1046 interphase nuclei were scored. Interphase FISH result showed 121 (11.6%) nuclei with hemizygous deletion for 22q11.2 locus and 925 (88.4%) nuclei with normal diploid state. About 15% metaphase also showed hemizygous deletion (Fig. 2C). Interphase FISH was also carried out on buccal cells and urinary cells to find out whether mosaicism restricted to blood or generalized. Mosaicism was confirmed in all three types of cells (Table 1). Parents were also screened for deletion and results were negative.

## Discussion and conclusion

In this report we have presented two cases of mosaic 22q11.2 microdeletion syndrome. Both of our cases had tetralogy of Fallot along with facial dysmorphism despite low level of deleted cells (~15%) in case 2. Mosaicism in 22q11.2 microdeletion syndrome is rare and only a few cases [Table 2] have been described in literature [7-9,12] with variable manifestation viz. early death [8,9] to asymptomatic carrier parents [7,9]. Mosaic full monosomy of chromosome 22 that may present similar to 22q11.2 microdeletion syndrome is also rare [Table 2] and reported few occasions [13-17] with variable manifestation. Similarly, reports of mosaic 22q11.2 deletion with duplication also have been described in the literature on 2 occasions [Table 2; [18,19]]. Chen et al. (2004) [7] described a fetus with mosaicism for 22q11.2 hemizygous microdeletion (a ratio of 43 deleted: 57 normal) on amniocytes using FISH technique. The case had cardiac malformation typical of tetralogy of Fallot along with thymic hypoplasia. The father of fetus also had mosaic 22q11.2 microdeletion (a ratio of 19 deleted: 81 normal) however completely asymptomatic. Patel et al. (2006) [9] reported another case of mosaicism for 22q11.2 microdeletion in fetal cardiac tissue as well in maternal lymphocytes (10%) by FISH analysis. Similarly, Consevage et al 1996 [8] and Hatchwell et al 1998 [12] reported mosaic 22q11.2 microdeletion in lymphocytes and germ cells, respectively. Dempsey et al (2007) [18] and Blennow et al (2008) [19] reported two more cases of mosaicism. However, patients were mosaic for two abnormal cell lines instead of normal/abnormal cell lines: a deletion [del(22)(q11.2q11.2)] and duplication [dup(22)(q11.2q11.2)].

**Table 2:** Published reports on mosaic 22q11.2 microdeletion and mosaic monosomy 22

**SN**	**References**	**Case description in brief**	**Method used**	**Results**
		***Mosaic 22q11.2 deletion***		
1	Chen et al 2004 [7]	Fetus with tetralogy of Fallot & thymic hypoplasia Father asymptomatic	Interphase FISH on amniocytes Interphase cells from cord blood Interphase FISH on lymphocytes	61% deleted interphase cells 43% deleted interphase cells 19% deleted interphase cells
2	Consevage et al 1996 [8]	Female newborn with facial dysmorphism, hypoplastic left heart syndrome & growth retardation	Metaphase FISH in lymphocytes	16% metaphases with deletion
3	Patel et al 2006 [9]	Missed abortion at 16 weeks gestation Asymptomatic carrier mother	Interphase FISH on heart tissue Lymphocytes	Cells with & without deletions 10% deleted cells
4	Hatchwell et al 1998 [12]	Mother with 2 affected & one unaffected child	FISH & haplotype analysis	Germ line mosai-cism in mother
		***Mosaic monosomy 22***		
5	Pinto-Escalante et al 1998 [13]	Dysmorphism, joint contracture, scleroderma, hypertrichosis, hypertonicity, etc	Karyotype from lymphocyte culture	15% monosomic cells
6	Verloes A et al 1987 [14]	A child with mild facial dysmorphism and mentally deficiency	Karyotype on lymphocytes & fibroblast cells	10.5% & 8.3% monosomic cells, respectively
7	Sabui and Chakrobarty 1997 [15]	Female child with facial dysmorphism & failure to thrive including developmental delay	Karyotype on lymphocytes	70% monosomic cells
8	Lewinsky et al 1990 [16]	Fetal gastroschisis with absent cerebral diastolic flow	Karyotype	Mosaic monosomy 22
9	Moghe et al 1981 [17]	Male child with facial dysmor-phism, psychomotor retardation, hypotonia & syndactyly	Karyotype on lymphocytes	25% monosomic cells
		***Mosaic 22q11.2 deletion & duplication***		
10	Dempsey et al 2007 [18]	Twin newborn, one normal & other with cardiac malformation as well as hypocalcemia, later delayed development and recurrent infections	FISH on metaphase of lymphocytes FISH on metaphase of skin fibroblasts FISH on interphase skin fibroblast cells	Deletion in 55% cells; duplication in 45% cells Deletion in 86% cells; duplication in 14% cells 80% deleted cells
11	Blennow et al. 2008 [19]	Girl with dysmorphism but no cardiac malformation	Lymphocytes	Deletion in 70% cells; duplication in 100% cells

Due to variable manifestation from case to case or familial transmission with this syndrome many authors were looked for mosaicism, including tissue specific. However, outcome were contradictory; some had proven (cardiac tissue) [9] or disproven [5,20,21] while others suspected for gonadal mosaicism [22,23] with different laboratory approaches. Ideal laboratory approach to diagnose mosaicism should be interphase FISH as other method, including QF PCR/MLPA can not detect low level mosaicism due to the presence of a normal cell line, which would mask the appearance of deleted 22q11.2 region. Since interphase FISH analysis can assess large numbers of individual cells very quickly this technique can pick up even low level of mosaicism. Our second case emphasizes the importance of interphase FISH in the diagnosis of low level of mosaicism despite strong clinical manifestations. To establish a mosaicism, as we followed & confirmed (on metaphase as well as with another probe), a repeat blood sample should always be analyzed along with other tissues viz. buccal cells (ectodermal origin) &/or urine cells (endodermal origin). However, one should take precaution before diagnosing a mosaicism that hemizygous signal drop is frequent with interphase FISH (also influenced by type of tissue and probes) [24,25]. In our hand 22q11.2 locus specific probe (RP5-882J5 & CTA-154H4) provides hemizygous results in 1.76% interphase lymphocyte nuclei of normal individuals and we had taken care before diagnosing low level mosaicism (i.e., also confirmed by metaphase FISH & second adjacent probe at 22q11.21 locus i.e., CTA-154H4 BAC clone; metaphase FISH does not give rise to hemizygous deletion in normal controls).

We also report on the association of trigonocephaly, one type of craniosynostosis, in one child with the mosaic 22q11.2 microdeletion. Yamamoto et al. (2006) [10] reported first time one patient with 22q11.2 microdeletion and craniosynostosis of the metopic suture leading to trigonocephaly. They suggested craniosynostosis of the metopic suture might be a minor complication, although coincidental occurrence cannot be ruled out. Here, we have encountered with another patient of 22q11.2 microdeletion (mosaic) with trigonocephaly derived from craniosynostosis of the metopic suture. This is the second report of a relationship between microdeletion 22q11.2 and trigonocephaly. Although trigonocephaly is rare with 22q11.2 microdeletion, it is commonly seen with monosomy 9p [26]. However, other form of craniosynostosis with 22q11.2 microdeletion syndrome is not so rare and reported in some previous occasions [27-31]. Ryan et al. (1997) [31] described five patients of microdeletion 22q11.2 syndrome with craniosynostosis without description of its type. McDonald-McGinn et al. (2005) [30] reported on the presence of craniosynostosis in another four patients with the 22q11.2 microdeletion syndrome. Similarly, De Silva et al. (1995), Karteszi et al. (2005), Dean et al. (1998), etc [27-29] reported some more cases of craniosynostosis in patient with microdeletion 22q11. In light of previous repeated reports of the association, we assume that craniosynostosis including trigonocephaly may be a rare manifestation of the 22q11.2 microdeletion syndrome. However, it seems that 12–15% mosaicism for 22q11.2 microdeletion may not solely account for the condition and the possibility of another undetected condition can not be ruled out. Craniosynostosis is also commonly seen with other chromosome deletions viz., with chromosome 15q (del(15)(q15q22.1) [32]; with chromosome 7p- [33-36] and 2q- [37]. Familial transmission of a 22q11.2 microdeletion accounts for 8 to 25% of the cases [38,39] and parents with deletions may be asymptomatic or mildly affected. However, none of the parents in our cases were carrier for the microdeletions.

We conclude that mosaic 22q11.2 microdeletion is not very rare if investigated with interphase FISH, can present as severe phenotype even in the presence of low level of deleted cells and trigonocephaly/craniosynostosis may be a rare manifestation.

## Competing interests

The authors declare that they have no competing interests.

## Authors' contributions

AH formulated activity plan, checked results & interpretated results. He also had reviewed clinical findings, prepared manuscript and responded to the quarries of reviewers. He will be the guarantor of the manuscript. MJ carried out all FISH related activity under guidance of AH. MK and NG were involved in clinical suspicion of the disease and management of the cases. All authors read and approved final manuscript.

## Consent

Written informed consent was obtained from the parent of patients for publication with any accompanying images. A copy of the written consent is available for review by the Editor-in-Chief of this journal [vide additional files 1-2]

## Supplementary Material

Additional File 1 **Consent form 1**. Click here for file

Additional File 2 **Consent form 2**. Click here for file

## References

[B1] Oskarsdottir S, Vujic M, Fasth A (2004). Incidence and prevalence of the 22q11 deletion syndrome: a population-based study in Western Sweden. Arch Dis Child.

[B2] Franke UC, Scambler PJ, Loffler C, Lons P, Hanefeld F, Zoll B, Hansmann I (1994). Interstitial deletion of 22q11 in DiGeorge syndrome detected by high resolution and molecular analysis. Clin Genet.

[B3] Park IS, Ko JK, Kim YH, Yoo HW, Seo EJ, Choi JY, Gil HY, Kim SJ (2007). Cardiovascular anomalies in patients with chromosome 22q11.2 deletion: a Korean multicenter study. Int J Cardiol.

[B4] Mantripragada KK, Tapia-Paez I, Blennow E, Nilsson P, Wedell A, Dumanski JP (2004). DNA copy-number analysis of the 22q11 deletion-syndrome region using array-CGH with genomic and PCR-based targets. Int J Mol Med.

[B5] Jianrong L, Yinglong L, Xiaodong L, Cuntao Y, Bin C, Bo W (2006). 22q11.2 deletion mosaicism in patients with conotruncal heart defects. Birth Defects Res A Clin Mol Teratol.

[B6] Jalali GR, Vorstman JA, Errami A, Vijzelaar R, Biegel J, Shaikh T, Emanuel BS (2008). Detailed analysis of 22q11.2 with a high density MLPA probe set. Hum Mutat.

[B7] Chen CP, Chern SR, Lee CC, Lin SP, Chang TY, Wang W (2004). Prenatal diagnosis of mosaic 22q11.2 microdeletion. Prenat Diagn.

[B8] Consevage MW, Seip JR, Belchis DA, Davis AT, Baylen BG, Rogan PK (1996). Association of a mosaic chromosomal 22q11 deletion with hypoplastic left heart syndrome. Am J Cardiol.

[B9] Patel ZM, Gawde HM, Khatkhatay MI (2006). 22q11 microdeletion studies in the heart tissue of an abortus involving a familial form of congenital heart disease. J Clin Lab Anal.

[B10] Yamamoto T, Sameshima K, Sekido K, Aida N, Matsumoto N, Naritomi K, Kurosawa K (2006). Trigonocephaly in a boy with paternally inherited deletion 22q11.2 syndrome. Am J Med Genet A.

[B11] Halder A, Fauzdar A (2007). Potential use of blood, buccal and urine cells for rapid noninvasive diagnosis of suspected aneuploidy using FISH. J Clin & Diagn Res.

[B12] Hatchwell E, Long F, Wilde J, Crolla J, Temple K (1998). Molecular confirmation of germ line mosaicism for a submicroscopic deletion of chromosome 22q11. Am J Med Genet.

[B13] Pinto-Escalante D, Ceballos-Quintal JM, Castillo-Zapata I, Canto-Herrera J (1998). Full mosaic monosomy 22 in a child with DiGeorge syndrome facial appearance. Am J Med Genet.

[B14] Verloes A, Herens C, Lambotte C, Frederic J (1987). Chromosome 22 mosaic monosomy (46,XY/45,XY,-22). Ann Genet.

[B15] Sabui TK, Chakraborty AK (1997). Monosomy 22 Mosaicism. Indian Pediatrics.

[B16] Lewinsky RM, Johnson JM, Lao TT, Winsor EJ, Cohen H (1990). Fetal Gastroschisis associated with monosomy 22 mosaicism and absent cerebral diastolic flow. Prenatal Diagnosis.

[B17] Moghe MS, Patel ZM, Peter JJ, Ambani LM (1981). Monosomy 22 with mosaicism. J Med Genet.

[B18] Dempsey MA, Schwartz S, Waggoner DJ (2007). Mosaicism del(22)(q11.2q11.2)/dup(22)(q11.2q11.2) in a patient with features of 22q11.2 deletion syndrome. Am J Med Genet A.

[B19] Blennow E, Lagerstedt K, Malmgren H, Sahlén S, Schoumans J, Anderlid B (2008). Concurrent microdeletion and duplication of 22q11.2. Clin Genet.

[B20] Rauch A, Hofbeck M, Cesnjevar R, Koch A, Rauch R, Buheitel G, Singer H, Weyand M (2004). Search for somatic 22q11.2 deletions in patients with conotruncal heart defects. Am J Med Genet A.

[B21] Vincent MC, Heitz F, Tricoire J, Bourrouillou G, Kuhlein E, Rolland M, Calvas P (1999). 22q11 deletion in DGS/VCFS monozygotic twins with discordant phenotypes. Genet Couns.

[B22] Kasprzak L, Der Kaloustian VM, Elliott AM, Shevell M, Lejtenyi C, Eydoux P (1998). Deletion of 22q11 in two brothers with different phenotype. Am J Med Genet.

[B23] Sandrin-Garcia P, Macedo C, Martelli LR, Ramos ES, Guion-Almeida ML, Richieri-Costa A, Passos GA (2002). Recurrent 22q11.2 deletion in a sib ship suggestive of parental germ line mosaicism in velocardiofacial syndrome. Clin Genet.

[B24] Iourov IY, Vorsanova SG, Yurov YB (2006). Intercellular Genomic (Chromosomal) Variations Resulting in Somatic Mosaicism: Mechanisms and Consequences. Current Genomics.

[B25] Iourov IY, Vorsanova SG, Yurov YB (2006). Chromosomal variation in mammalian neuronal cells: known facts and attractive hypotheses. Int Rev Cytol.

[B26] Azimi C, Kennedy SJ, Chitayat D, Chakraborty P, Clarke JT, Forrest C, Teebi AS (2003). Clinical and genetic aspects of trigonocephaly: A study of 25 cases. Am J Med Genet Part A.

[B27] De Silva D, Duffty P, Booth P, Auchterlonie I, Morrison N, Dean JC (1995). Family studies in chromosome 22q11 deletion: further demonstration of phenotypic heterogeneity. Clin Dysmorphol.

[B28] Dean JC, De Silva DC, Reardon W (1998). Craniosynostosis and chromosome 22q11 deletion. J Med Genet.

[B29] Karteszi J, Kress W, Szasz M, Czako M, Melegh B, Kosztolanyi GY, Morava E (2004). Partial craniosynostosis in a patient with deletion 22q11. Genet Couns.

[B30] McDonald-McGinn DM, Gripp KW, Kirschner RE, Maisenbacher MK, Hustead V, Schauer GM, Keppler-Noreuil KM, Ciprero KL, Pasquariello PJr, LaRossa D (2005). Bartlett S.P., Whitaker L.A., Zackai E.H., Craniosynostosis: another feature of the 22q11.2 deletion syndrome. Am J Med Genet A.

[B31] Ryan AK, Goodship JA, Wilson DI, Philip N, Levy A, Seidel H, Schuffenhauer S, Oechsler H, Belohradsky B, Prieur M, Aurias A, Raymond FL, Clayton-Smith J, Hatchwell E, McKeown C, Beemer FA, Dallapiccola B, Novelli G, Hurst JA, Ignatius J, Green AJ, Winter RM, Brueton L, Brondum-Nielsen K, Stewart F, Van Essen T, Patton M, Paterson J, Scambler PJ (1997). Spectrum of clinical features associated with interstitial chromosome 22q11 deletions: A European collaborative study. J Med Genet.

[B32] Fukushima Y, Wakui K, Nishida T, Nishimoto H (1990). Craniosynostosis in an infant with an interstitial deletion of 15q [46,XY,del(15)(q15q22.1)]. Am J Med Genet.

[B33] Aughton DJ, Cassidy SB, Whiteman DA, Delach JA, Guttmacher AE (1991). Chromosome 7p-syndrome: craniosynostosis with preservation of region 7p2. Am J Med Genet.

[B34] Gong BT, Norwood TH, Hoehn H, McPherson E, Hall JG, Hickman R (1976). Chromosome 7 short arm deletion and craniosynostosis. A 7p-syndrome. Hum Genet.

[B35] Motegi T, Ohuchi M, Ohtaki C, Fujiwara K, Enomoto S, Hasegawa T, Kishi K, Hayakawa H (1985). A craniosynostosis in a boy with a del(7)(p15.3p21.3): assignment by deletion mapping of the critical segment for craniosynostosis to the mid-portion of 7p21. Hum Genet.

[B36] Shetty S, Boycott KM, Gillan TL, Bowser K, Parboosingh JS, McInnes B, Chernos JE, Bernier FP (2007). Cytogenetic and molecular characterization of a de-novo cryptic deletion of 7p21 associated with an apparently balanced translocation and complex craniosynostosis. Clin Dysmorphol.

[B37] Nixon J, Oldridge M, Wilkie AO, Smith K (1997). Interstitial deletion of 2q associated with craniosynostosis, ocular coloboma, and limb abnormalities: cytogenetic and molecular investigation. Am J Med Genet.

[B38] Digilio MC, Angioni A, De Santis M, Lombardo A, Giannotti A, Dallapiccola B, Marino B (2003). Spectrum of clinical variability in familial deletion 22q11.2: from full manifestation to extremely mild clinical anomalies. Clin Genet.

[B39] Driscoll DA, Salvin J, Sellinger B (1993). Prevalence of 22q11 microdeletions in DiGeorge and velo-cardio-facial syndromes: implications for genetic counseling and prenatal diagnosis. J Med Genet.

