# Supplementary figures and images for: Mosaic 22q11.2 microdeletion syndrome: diagnosis and clinical manifestations of two cases
Source: Mol Cytogenet. 2008 Aug 10;1:18. doi: 10.1186/1755-8166-1-18 (PMC2527005; doi:10.1186/1755-8166-1-18)

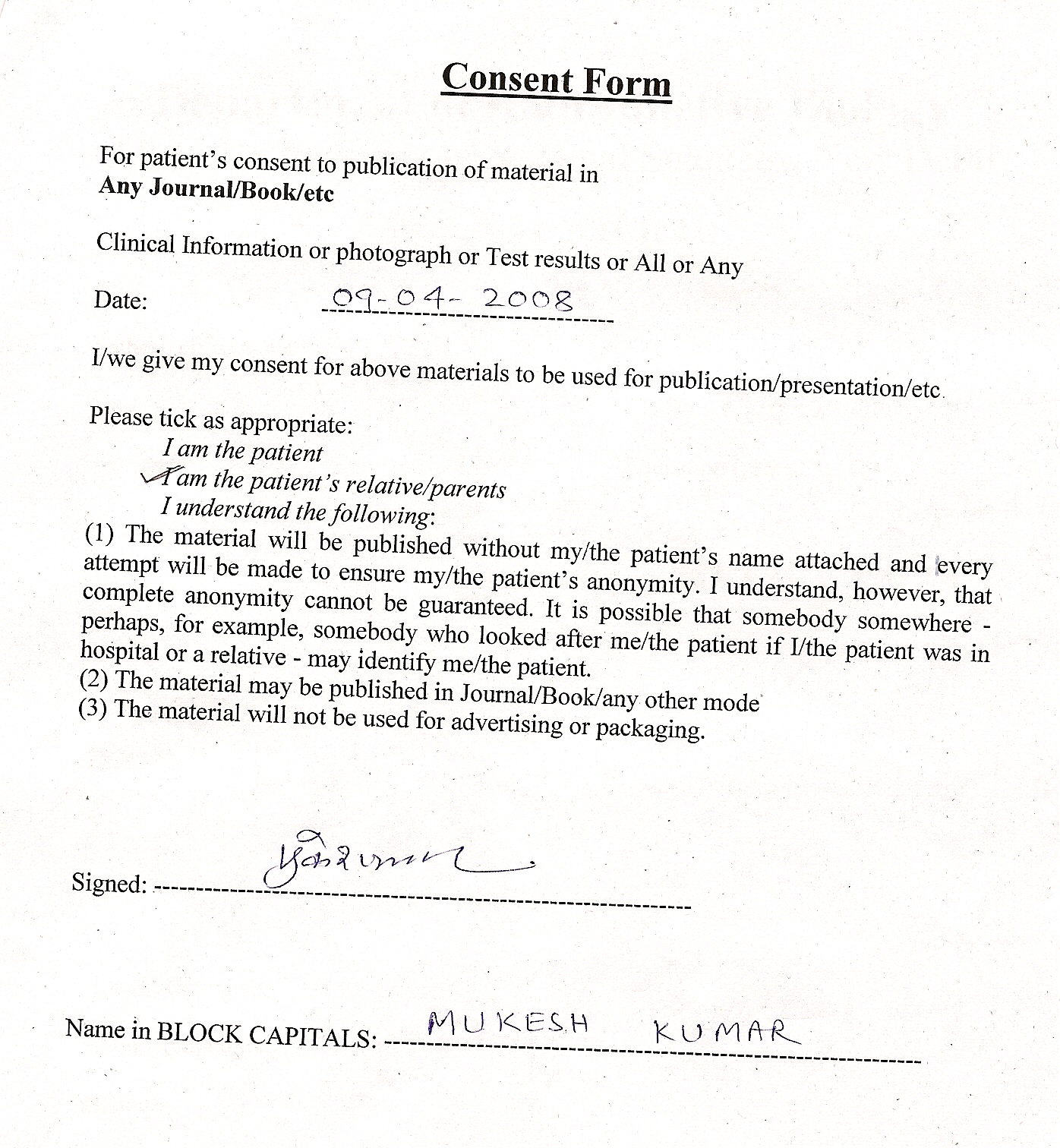

Supplement: Additional File 1 — Consent form 1. [file 1755-8166-1-18-S1.jpeg]

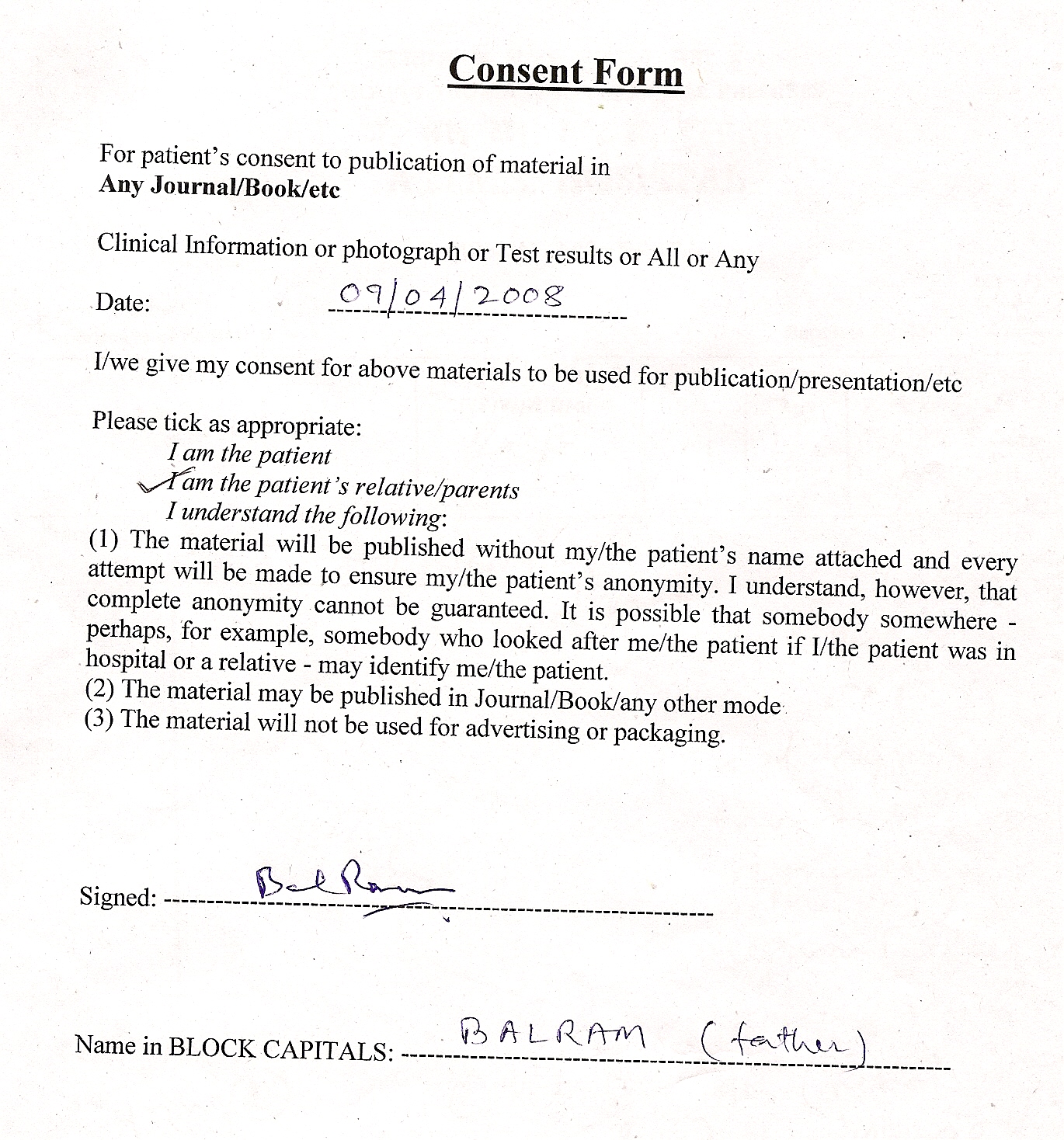

Supplement: Additional File 2 — Consent form 2. [file 1755-8166-1-18-S2.jpeg]
